# From End-of-Life Care to Improved Quality of Life and Better Prognosis by Using Vericiguat: A Case Report From Costa Rica

**DOI:** 10.7759/cureus.39570

**Published:** 2023-05-27

**Authors:** Mario Speranza-Sánchez, Esteban Zavaleta-Monestel, Sonia Sancho-Zumbado, Sebastián Arguedas-Chacón, Alonso Quirós-Romero

**Affiliations:** 1 Heart Failure Program, Hospital Clínica Bíblica, San José, CRI; 2 Pharmacy Department, Hospital Clínica Bíblica, San José, CRI; 3 Faculty of Pharmacy, University of Costa Rica, San José, CRI

**Keywords:** left ventricular ejection fraction, valvulopathy, lvef (left ventricular ejection fraction), chronic kidney failure, soluble guanylyl cyclase, heart failure with reduced ejection fraction

## Abstract

In this case report, we present the evolution of a heart failure with reduced ejection fraction (HFrEF) patient who was set to receive end-of-life care but demonstrated improvement following treatment with vericiguat in combination with foundational therapy. Vericiguat is a novel soluble guanylate cyclase stimulant that has been proven helpful for treating decompensated heart failure with HFrEF, decreasing hospitalization rates and mortality of cardiovascular causes. This medication is currently indicated in patients who require IV diuretics administration or hospitalization due to decompensated heart failure. This is a case study of a 62-year-old woman with dilated heart failure and reduced left ventricular ejection fraction (LVEF), who was a wheelchair user due to severe cardiovascular symptoms and various comorbidities, who was referred to our heart failure program for treatment. Despite previous treatment, the patient experienced persistent cardiovascular symptoms and required palliative care. After optimizing the foundational therapy, the patient's condition improved but continued to require hospitalization. Vericiguat was initiated as an add-on. After six months, the patient's LVEF improved by 9%, and she is now asymptomatic with a considerable decrease in pro-B-type natriuretic peptide levels and is wheelchair independent due to enhance exercise resistance. However, the echocardiogram revealed a progression in the dysfunction of both the mitral and aortic valves. The patient's renal function and quality of life scores also changed over time. Vericiguat therapy, as an adjunct to foundational therapy, improved exercise tolerance and symptom relief. However, further investigation is necessary to assess the effects of vericiguat on renal function and disease progression in individuals with HFrEF.

## Introduction

Heart failure (HF) is a chronic condition that poses a significant burden on healthcare systems and affects over 60 million people worldwide, resulting in frequent hospitalizations and reduced quality of life for those affected [1]. Despite advancements in treatment options, there remains an unmet need for effective therapies that can improve clinical outcomes and mitigate the economic impact of this disease. In recent years, research has focused on identifying novel pharmacologic agents that can control disease progression, reduce mortality and hospitalization rates, and improve patients' overall quality of life [2].

Current guidelines for managing heart failure with reduced ejection fraction (HFrEF) recommend a foundational quadruple therapy that includes beta blockers, sodium-glucose cotransporter-2 (SGLT2) inhibitors, angiotensin receptor-neprilysin inhibitors (ARNIs), and mineralocorticoid receptor antagonists (MRAs) [3]. This approach has been shown to extend life expectancy by a median of six years compared to more limited regimens [4]. However, despite the use of this therapy, HFrEF patients may still experience frequent decompensation and hospitalization events [5].

One of the recently identified therapeutic targets is the nitric oxide (NO)/cyclic guanosine monophosphate (cGMP) signaling pathway. This pathway employs nitric oxide to activate soluble guanylate cyclase (sGC), which then generates cGMP. The resulting cGMP triggers smooth muscle cell relaxation and a reduction in hypertrophy, inflammation, and fibrosis. SGC stimulators like vericiguat have shown promise in the treatment of decompensated HFrEF [6,7].

Studies have demonstrated that vericiguat can reduce the risk of cardiovascular death or hospitalization in patients with HFrEF [8]. As of January 21, 2021, the Food and Drug Administration (FDA) has approved the use of vericiguat to treat HFrEF [9]. However, it should be noted that the medication does not halt disease progression. In addition, the impact of vericiguat in combination with SGLT2 inhibitors is not yet established. Nonetheless, incorporating vericiguat into the foundational therapy may offer supplementary advantages [5,10].

In this case report, we present the exceptional evolution of an HFrEF patient who was set to receive end-of-life care but demonstrated remarkable improvement following treatment with vericiguat in combination with the foundational therapy.

## Case presentation

A 62-year-old woman with HFrEF, a reduced left ventricular ejection fraction (LVEF) of 15%, pro-B-type natriuretic peptide (pro-BNP) baseline levels of 18,244 pg/mL (normal range < 125 pg/mL), and New York Heart Association (NYHA) class III, who was a wheelchair user due to severe cardiovascular symptoms, was referred to the Heart Failure Program (HFP) for assessment and treatment. Her caretaker had to transport her due to the severity of her condition. The patient had a history of grade III cardiomegaly, cardio-renal syndrome type II with chronic renal failure stage IV, dyslipidemia, peripheric arterial disease, chronic obstructive pulmonary disease, anemia of renal cause, and resolved hepatitis C infection.

The patient, under the care of her initial medical center, was administered the following treatment regimen: irbesartan at a dosage of 300 mg once daily, lovastatin at a dosage of 40 mg once daily, furosemide at a dosage of 80 mg twice daily, acetylsalicylic acid at a dosage of 100 mg once daily, carvedilol at a dosage of 25 mg twice daily, and spironolactone at a dosage of 25 mg once daily. She remained weak and experienced persistent cardiovascular symptoms. Eventually, palliative care was provided, but the severity of the patient's condition caused her to be discharged and sent home for end-of-life care.

Upon reaching the HFP, the patient reported experiencing dyspnea and palpitations. As the patient had not been previously classified under the NYHA functional classification system, she was assigned an NYHA class III status at that time. An EKG was performed releveling a left bundle branch block. At the time of the patient's admission, prioritizing treatment optimization was of utmost importance. Foundational therapy was completed, switching carvedilol to metoprolol 100 mg twice a day, initiating dapagliflozin 10 mg once daily, changing irbesartan to sacubitril/valsartan 24.3/25.7 mg twice daily, maintaining spironolactone 25 mg once daily, and also adding ivabradine 2.5 mg twice a day. Despite these changes, the patient required an intravenous furosemide session shortly after her initial appointment due to fluid congestion. The patient's treatment plan was tailored to address her specific needs, with an emphasis on optimizing pharmacotherapy and managing symptoms of heart failure. Unfortunately, the patient's condition deteriorated and she needed to be hospitalized due to severe heart failure decompensation.

After being hospitalized, the patient's condition progressed to NYHA class II with the aid of foundational therapy. Additionally, there was a marginal improvement in the LVEF to 18%. As the patient met the inclusion criteria for the VICTORIA trial [8], efforts were made to initiate vericiguat therapy. The medication was titrated according to the manufacturer's recommendations, starting at 2.5 mg once daily and doubling it every two weeks until reaching the target dose of 10 mg once daily. The patient's systolic blood pressure was closely observed due to the potential for hypotension, but fortunately, she did not experience this adverse reaction. One month after initiating vericiguat as an add-on therapy, the patient's exercise tolerance improved substantially, no longer requiring a wheelchair for mobility and reporting no cardiovascular symptoms during exercise. However, the patient's anemia persisted and worsened, dropping hemoglobin levels to 10.4 g/dL. While vericiguat may have contributed to anemia as one of its adverse reactions, due to the patient's history of the condition and the worsening in renal function, it was more probable that anemia had a renal cause.

**Figure 1:**
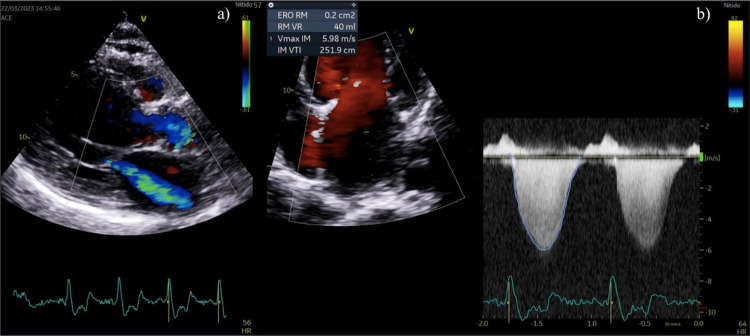
Retrograde blood flow due to mitral valve dysfunction during systole (a) and severity measurements of this valvulopathy (b)

**Figure 2:**
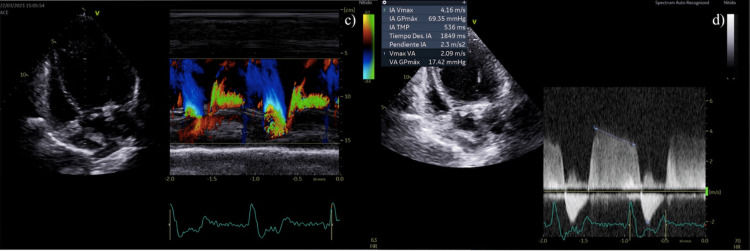
Color M mode of aortic regurgitation during diastole (c) and severity measurements of this valvulopathy (d)

In the latest echocardiogram taken after six months of vericiguat therapy, an improvement in LVEF was observed, reaching 24% with current pro-BNP levels of 7,866 pg/dL. However, the disease progression resulted in moderate mitral valve malfunction, as shown in Figure 1. Due to aortic atherosclerosis, the patient developed a mild aortic dysfunction, which is represented in Figure 2. Table 1 illustrates the evolution in renal function, cardiac function markers, and quality of life scores.

**Table 1:** Evolution of renal function, cardiac markers, and quality of life since the time of admission and current values with six months of vericiguat ^a^ Patient's evolution with foundational therapy. ^b^ Patient's evolution with foundational therapy plus vericiguat. Abbreviations: Cr, creatinine; CrCl, creatinine clearance; BUN, blood urea nitrogen; LVEF, left ventricular ejection fraction; Pro-BNP, pro-B-type natriuretic peptide; NYHA, New York Heart Association.

Parameter	Base value	Mid-term value^a^	Current value^b^
Cr	2.14 mg/dL	2.80 mg/dL	3.15 mg/dL
CrCl	26 mL/min/1.73 m^2^	19 mL/min/1.73 m^2^	16 mL/min/1.73 m^2^
BUN	69.9 mg/dL	94 mg/dL	71 mg/dL
LVEF	15%	18%	24%
Pro-BNP levels	18,244 pg/mL	-	7,866 pg/mL
NYHA class	III	II	I
Barthel score	75 pts	-	100 pts
Kansas score	30 pts	-	59 pts
Six-minute walk	-	-	360 m

## Discussion

The use of sGC stimulants, such as vericiguat, has been limited to the treatment of pulmonary arterial hypertension and chronic thromboembolic pulmonary hypertension. In recent years, however, these agents have emerged as promising add-on therapy for decompensated HFrEF [10,11]. NO, a potent vasodilator, is produced endogenously in the blood vessels and activates sGC, leading to the production of cGMP. This pathway mediates downstream effects throughout cGMP targets, resulting in vasodilation, inhibition of platelet aggregation, fibrosis, and smooth muscle proliferation. In the heart, natriuretic peptides activate the NO-sGC-cGMP pathway, leading to cardioprotective actions, such as natriuresis, improved diastolic relaxation, coronary blood flow, reduced inflammation, hypertrophy, and fibrosis [10-12]. However, in patients with HF, comorbidities and hypoperfusion contribute to oxidative stress and inflammation of the heart, which results in reduced signaling of the NO-sGC-cGMP pathway. Vericiguat could potentially have a pivotal role in managing this disease, as it boosts this pathway [7,10-12].

In the VICTORIA trial, vericiguat therapy was shown to reduce mortality from cardiovascular causes and hospitalization rates, thereby improving the patient's quality of life [8]. According to these outcomes, the case presented in this report also showed a remarkable improvement in symptoms and therefore, an improvement in quality of life and no hospital readmission since the vericiguat therapy was initiated. Before the optimized therapy, the patient was considered a palliative care candidate by several healthcare professionals, some even recommending discharge and ambulatory care. However, it should be noted that the patient was not receiving the foundational therapy for HFrEF before the HFP admission. Following therapy optimization, the patient's NYHA class eventually improved to class I. Therefore, not every aspect of her improvement can be attributed solely to vericiguat therapy. Nonetheless, the main change the patient experienced with vericiguat therapy was a significant improvement in exercise capacity, now independent from the use of a wheelchair.

In addition, the improvement in quality of life, the decrease in pro-BNP levels, and the increase in LVEF were also seen in the VICTORIA trial [8]. It is important to note that while these changes are promising and represent better quality of life and less symptomatology, they do not represent a halt in disease progression [7,8]. In the latest functionality tests and quality of life scores, she showed improvement, and now being able to walk, the patient reached a distance of 360 m in the six-minute walk, as shown in Table 1. Upon completion of the test, the patient did not exhibit any cardiovascular symptoms. However, the limited walking distance was attributed to acute pain in both legs. It is necessary to continue performing these tests to continue determining the evolution of the disease.

In end-stage renal failure, dialysis patients, and chronic kidney disease due to increased uremic nitrogen associated with poor protein metabolite clearance, it is possible to find uremic xerosis. As this patient reached blood urea nitrogen (BUN) peaks of 94 mg/dL, as shown in Table 1, uremic xerosis was developed, although fortunately, it did not evolve into uremic pruritus [13,14]. The optimization of the foundational therapy and the addition of vericiguat could have a possible positive effect on renal function, as indicated by a slight improvement in BUN levels. Additionally, the patient is currently not experiencing any symptoms of uremic xerosis. But despite this positive change, renal function did not improve in this patient, as the disease continued progressing. The renal function in vericiguat therapy is a particular point of interest as a reduced creatinine clearance of 15-30 mL/min/1.73 m^2^ was part of the inclusion criteria, but despite treating renal patients, evolution in this matter was not part of the VICTORIA trial outcomes [8]. Further studies are needed to determine the long-term effects of vericiguat therapy on renal function and disease progression in patients with HFrEF.

## Conclusions

SGC stimulants such as vericiguat have emerged as a promising therapy for decompensated HFrEF. The case presented in this report also showed significant improvement in symptoms and exercise capacity after initiating vericiguat therapy as an add-on therapy to the foundational therapy. Additional research is required to determine the impact of vericiguat treatment on both renal function and disease progression among HFrEF patients.
